# Target-small decoy search strategy for false discovery rate estimation

**DOI:** 10.1186/s12859-019-3034-8

**Published:** 2019-08-23

**Authors:** Hyunwoo Kim, Sangjeong Lee, Heejin Park

**Affiliations:** 1 Research Data Sharing Center, Korea Institute of Science and Technology Information, Daejeon, 34141 Republic of Korea; 2 Department of Computer Science, Hanyang University, Seoul, 06978 Republic of Korea

**Keywords:** Target-decoy search, Target-small decoy search, False discovery rate

## Abstract

**Background:**

One of the most important steps in peptide identification is to estimate the false discovery rate (FDR). The most commonly used method for estimating FDR is the target-decoy search strategy (TDS). While this method is simple and effective, it is time/space-inefficient because it searches a database that is twice as large as the original protein database. This inefficiency problem becomes more evident as protein databases get bigger and bigger. We propose a target-small decoy search strategy and present a rigorous verification that it reduces the database size and search time while retaining the accuracy of target-decoy search strategy (TDS).

**Results:**

We show that peptide spectrum matches (PSMs) obtained at 1% FDR in TDS overlap ~ 99% with those in our method. (Considering that 1% FDR is used, 99% overlap means our method is very accurate.) Moreover, our method is more time/space-efficient than TDS. The search time of our method is reduced to only 1/4 of that of TDS when UniProt and its 1/8 decoy database are used.

**Conclusions:**

We demonstrate that our method is almost as accurate as TDS and more time/space-efficient than TDS. Since the efficiency of our method is more evident as the database size increases, our method is expected to be useful for identifying peptides in proteogenomics databases constructed from inflated databases using genomic data.

**Electronic supplementary material:**

The online version of this article (10.1186/s12859-019-3034-8) contains supplementary material, which is available to authorized users.

## Background

Proteomics is a powerful technology in molecular cell biology. Proteins are identified by peptide sequences which are identified by tandem mass spectra (MS/MS). [1] One of the most important steps in peptide identification is to estimate the PSM/peptide-level false discovery rate (FDR). The commonly used methods for estimating FDR are the target-decoy search strategy (TDS) [2] and mixture model-based methods. [3, 4]

TDS is a method of FDR estimation using a decoy database and can estimate the number of false positives by doubling the number of selected decoy. While TDS is simple and effective, it is time/space-inefficient because it searches a database that is twice as large as the original protein database. This inefficiency problem becomes more evident as protein databases get bigger and bigger. For example, current proteogenomics requires searching a database constructed from enormous genomic data. [5–8] To resolve this inefficiency problem, several methods have been proposed to estimate FDR without the decoy database (only target database). [9, 10] However, none of the proposed methods is considered as accurate as TDS in estimating FDR, and thus TDS is still widely used.

There are several methods to construct decoy databases. They are either reversing, pseudo reversing, shuffling, or pseudo shuffling protein sequences. When reversing or pseudo reversing is used (Additional file 1: Method), the number of target false positives approximates to the number of decoy false positives, and thus the number of decoy matches and the number of target matches are used to estimate FDR. However, when shuffling or pseudo shuffling is used (Additional file 1: Method), the number of decoy false positives becomes bigger than the number of target false positives (Additional file 1: Figure S1). To compensate this, Elias and Gygi [2, 11] suggested a multiplicative factor that is multiplied to the number of decoy false positives to approximate it to the target false positives.

Our contribution is two-fold. First, we extend this multiplicative factor approach further to handle the cases when the sizes of target and decoy databases are different. Especially, we focus on the case when the decoy database is smaller than the target database which is named a target-small decoy search strategy. In our method, the decoy database size is reduced intentionally so that the search time is faster and the memory requirement is smaller than a normal TDS. (Note that we studied “unequal database sizes” which is different from “unequal number of unique peptides in equal database sizes” studied by Elias and Gygi.)

**Fig. 1:**
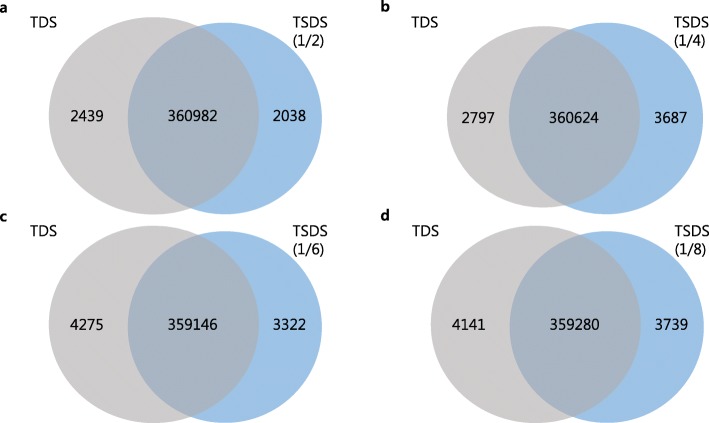
Comparison of PSMs between TDS and our method using Comet. (**a**) 1/2 decoy database; (**b**) 1/4 decoy database; (**c**) 1/6 decoy database; (**d**) 1/8 decoy database. Using the UniProt database

**Fig. 2:**
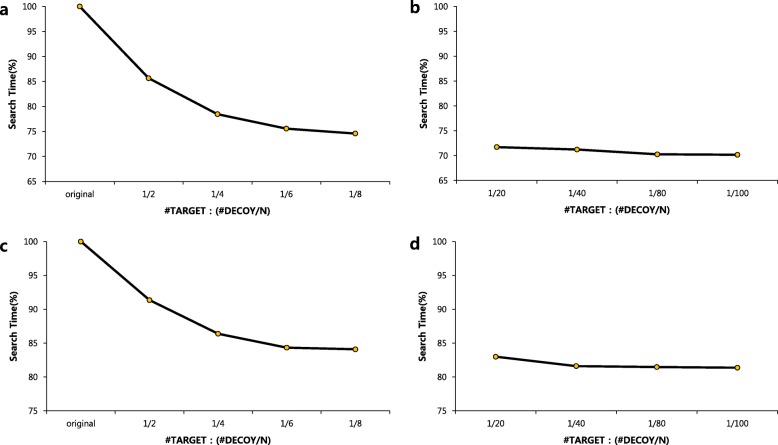
Comparison of search times for various decoy database sizes when the search time of the original decoy database is 100%. It shows that is little change in search time when the decoy database is reduced to 1/4 or less. #TARGET: (#DECOY/N) indicates the size at which the decoy database is reduced compared to the target. (**a**), (**b**) Using the UniProt database. (**c**), (**d**) Using the SwissProt database

Second, we present a rigorous verification that our method retains the accuracy of TDS. We show that peptide spectrum matches (PSMs) obtained at 1% FDR in TDS overlap 99% with those in our method. Considering that 1% FDR is used, 99% overlap means our method is very accurate. Our experiments show that a small decoy whose size is 1/N of the target database size retains the accuracy of normal TDS (Fig. 1) and, in addition, the search time is only 1/4 of that of TDS when UniProt and its 1/8 decoy database are used (Fig. 2a).

## Results

We compared our method with TDS using original HEK293 data set. This data set was searched by Comet with the following common high resolution parameter settings: precursor tolerance = 10 ppm, fragment tolerance = 0.02 Da, NTT = 2, maximum missed cleavages = 2, and fixed modification = Carbamidomethyl on cysteine. PSMs were obtained at 1% FDR using TDS and our method. Using UniProt and SwissProt databases, Fig. 1 and Additional file 1: Figure S2 compare the results of TDS with our method, respectively. Our method identified 98.7% ~ 99.3% PSMs identified by TDS. In addition, when the other 10 cell lines were searched using UniProt and SwissProt databases and their 1/8 decoy databases, respectively, our method identified 98.5% PSMs identified by TDS on average. (Additional file 1: Figures S3 and S4). We also compared our method with TDS using *Saccharomyces cerevisiae* dataset [12]. This data set was searched by Comet with the following common low resolution parameter settings: precursor tolerance = 10 ppm, fragment tolerance = 0.5 Da, NTT = 2, maximum missed cleavages = 2, and fixed modification = Carbamidomethyl on cysteine. Using UniProt *Saccharomyces cerevisiae* database, Additional file 1: Figure S5 compares the results of TDS with our method. Our method identified 98.7% PSMs identified by TDS on average.

We checked the variations of searching results using four UniProt human random decoy database. HEK293 data set was searched by Comet using four 1/8 decoy databases for UniProt database. Additional file 1: Figure S6 compares the results of TDS with our method and shows little variation. Our method identified 98.8% PSMs identified by TDS on average.

MSGF+ [13] was also run on UniProt database for HEK 293 data set with the following high resolution parameters: precursor tolerance = 10 ppm, fragment tolerance = 0.02 Da, NTT = 2, and fixed modification = Carbamidomethyl on cysteine. Additional file 1: Figure S7 compares the results of TDS with Method of our method. Our method identified 99.8% PSMs identified by TDS.

Furthermore, search time of peptide identification is proportional to the size of the each database (Fig. 2). For example, search time of our method is only 1/4 of that of TDS when UniProt and its 1/8 decoy database is used on a machine with an Intel Xeon CPU E5–2609 (1.90GHz) and 36GB of RAM using 6 threads.

## Conclusions

In summary, we demonstrate that our method is almost as accurate as TDS and more time/space-efficient than TDS. Since the efficiency of our method is more evident as the database size increases, our method is expected to be useful for identifying peptides in proteogenomics databases constructed from inflated databases using genomic data.

## Discussion

We performed additional search using smaller decoy databases than 1/8 decoy database. HEK293 data set was searched by Comet using 1/20, 1/40, 1/80, and 1/100 decoy databases for UniProt and SwissProt databases, respectively. Our method identified 98.6% PSMs identified by TDS on average (Additional file 1: Figure S8 and S9). It means that FDR can be estimated using smaller decoy databases than 1/8 decoy database. However, the speedup for 1/100 decoy is similar to the speedup for 1/8 decoy.

It should be noted that a small decoy database is generated by a random selection and it is different for every run. Of course, this does not influence the accuracy as shown in this paper. However, if some researchers are in a situation in which the decoy database should be fixed, they can store and reuse the first generated small decoy database, which is not a big overhead because the decoy database is small.

## Methods

To estimate the FDR using TDS, 1) TDS considers the PSM hit in the decoy database as incorrect and 2) the number of false positive at target PSM is assumed almost same as number of decoy hit. TDS proposed two assumptions and showed that these two assumptions are reasonable. We modify two assumptions to use a small decoy database and then we propose a method for estimating the FDR. We show that modified assumptions is reasonable and then discuss to method to estimate the FDR using a small decoy database.

Two assumptions are used to estimate the FDR by our method:

Assumption 1: Target and decoy databases do not overlap;

Assumption 2-1: The ratio of decoy to target false positives (***FPRatio***) is specific.

Assumption 2-2: *FPRatio* is almost the same as the ratio of decoy to target unique peptides (***UPRatio***) in a given database.

**Fig. 3:**
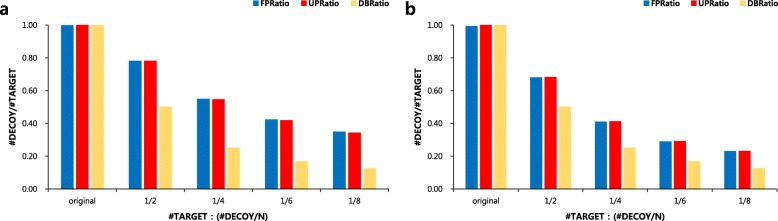
Comparison of ratios among *FPRatio* (blue bars), *UPRatio* (red bars) and *DBRatio* (yellow bars). (**a**) Using the UniProt database. (**b**) Using the SwissProt database. #DECOY/#TARGET indicates the ratio of decoy to target for each method

Assumption 1 is identical to TDS (It is shown in Additional file 1: Methods). Assumption 2-1 and 2-2 are important because these enable estimating the FDR with a small decoy database. This is because the probability is that a false positive appears in the target PSM should be obtained by the decoy PSM. It should be noted that *FPRatio* is not similar to ***DBRatio***, the ratio of decoy to target database sizes, but it is similar to *UPRatio*.(Fig. 3) Thus, once *UPRatio* is obtained, FDR estimation using our method is easy.

### Data set

An MS/MS data set from 11 human cell lines (A549, GAMG, HEK293, HeLa, HepG2, Jurkat, K562, LnCap, MCF7, RKO, and U2OS, each 3 replicates) [14] was obtained by an LTQ-Orbitrap Velos mass spectrometer (Thermo Fisher Scientific, Bremen, Germany). An MS/MS data set from *Saccharomyces cerevisiae* [12] was obtained by Orbitrap Fusion (Thermo Fisher Scientific, Bremen, Germany). The higher-energy collisional dissociation (HCD) method was used for peptide fragmentation. HCD can efficiently fragment peptide ions for high-accuracy and full-mass-range tandem mass spectrometry. Additional file 1: Table S1 shows the number of spectrum in human cell lines and *Saccharomyces cerevisiae*.

### Small decoy database construction

We used three databases: 1) The target database consists of the UniProt human protein database (v201601; 172,121 entries) and 179 common contaminants. 2) The target database consists of the SwissProt human protein database (v201601, 42,123 entries) and 179 common contaminants. 3) The target database consists of the Uniprot *Saccharomyces cerevisiae* protein database (v201901, 6721 entries) and 179 common contaminants. In these target databases, decoy databases are constructed by reversing the target database and by picking random proteins according to the ratio of small-decoy to target database sizes (Additional file 1: Table S2 and S3).

### Assumption 2

To validate Assumption 2, we used HEK293 data set and UniProt human protein database.

### Assumption 2-1: The ratio of decoy to target false positives (*FPRatio*) is specific.

Actually, Assumption 2-1 presented in this paper is a generalization of assumption 2 presented by Elias and Gygi. [2] They considered only the case when the decoy database size is the same as the target database size but we consider the general cases when the decoy database size can be smaller than the target database size. Thus, assumption 2-1 is validated by shifting precursor mass method which is suggested by Elias and Gygi and is to shift precursor masses of tandem mass spectra. [2] HEK293 data set was used by shifting precursor masses of tandem mass spectra by 10 Da (Dalton). Because shifted precursor masses are not real precursor masses, identified PSMs (peptide spectrum matches) can be considered as incorrect and false positives. Shifted HEK293 data were searched by Comet [15] with the following high resolution parameter settings: precursor mass tolerance = 10 ppm, fragment tolerance = 0.02 Da, number of tryptic termini (NTT) = 2, maximum missed cleavage = 2, and fixed modification of carbamidomethyl on Cys. Comet search was done against the five databases consisting of the target database and five decoy databases with different sizes. The sizes of decoy databases are 1, 1/2, 1/4, 1/6, and 1/8 of the target database size, respectively.

**Fig. 4:**
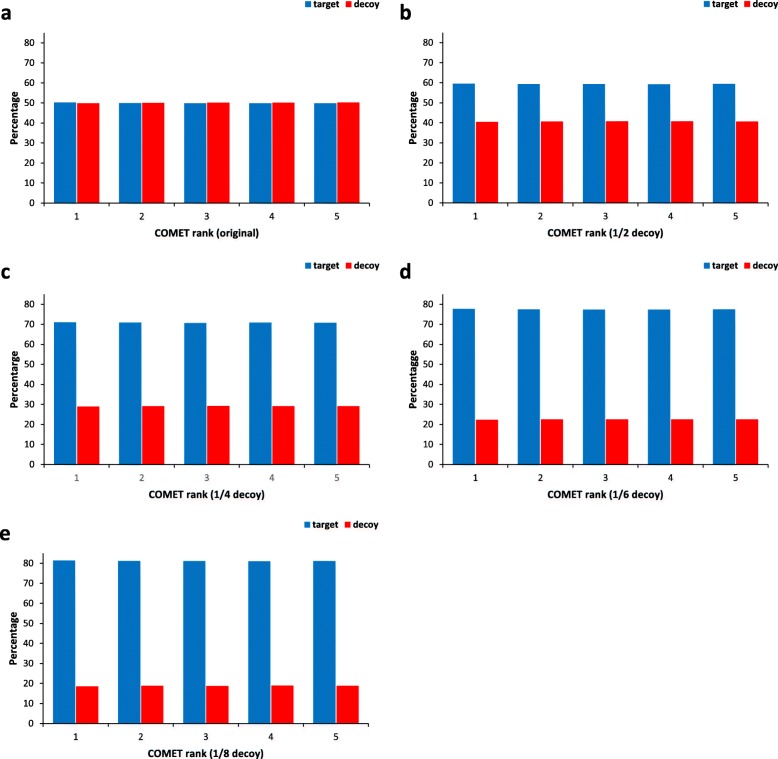
The percentages of target/decoy PSMs among the ranks 1–5 PSMs in the Comet results using shifted HEK293 data. Blue bars represent the percentage of target PSMs and red bars represent that of decoy PSMs. (**a**) Original decoy database; (**b**) 1/2 decoy database; (**c**) 1/4 decoy database; (**d**) 1/6 decoy database; (**e**) 1/8 decoy database. Using the UniProt database

Figure 4 shows the ratio of decoy to target false positives for each rank 1 to 5 obtained from 624,108 searches by Comet. The ratios are almost the same regardless of ranks in each small decoy case. For example, for a 1/8-sized small decoy (Fig. 4e), they are all 0.34 for each rank 1–5. Furthermore, the ratios of 3 replicates are almost the same (Additional file 1: Fig. S10). Conclusively, Assumption 2-1 is valid and FDR can be estimated once the ratio of decoy to target false positives is calculated.

### Assumption 2-2: *FPRatio* is almost the same as the ratio of decoy to target unique peptides (*UPRatio*) in a given database.

Assumption 2-2 is validated by showing that *FPRatio* is almost the same as *UPRatio*. Unique peptides are achieved using the following parameter settings: maximum missed cleavage site = 2, NTT = 2, minimum peptide length = 6, and maximum peptide length = 45. We propose to calculate *UPRatio* for a given database.

Method: Compute the ratio of decoy to target unique peptides

$$ UPRatio=\frac{Dmass\left({n}_d\right)}{Tmass\left({n}_t\right)} $$

Where *n*_*t*_ and *n*_*d*_ are the largest precursor masses in the target and decoy unique peptides, respectively.

When *UPRatio* is computed using this method above, these two ratios, *UPRatio* and *FPRatio*, are almost identical (Fig. 3). Hence, by using this method, *FPRatio* can be easily approximated.

Furthermore, Additional file 1: Fig. S11 shows the ratio of decoy to target unique peptides at all mass windows for candidate peptides with precursor mass tolerance = 10 ppm. Each point represents the number of target unique peptides as its *x* axis and the number of decoy unique peptides as its *y* axis in a mass window. The slope of Additional file 1: Figure S11 is almost identical to the *UPRatio*. (Additional file 1: Figure S12).

### How to estimate FDR using small decoy databases

Target and decoy false positives are equally likely when using TDS. Generally, FDR is estimated in TDS using the following equation:

$$ {FDR}_{TDS}=\frac{\# Decoy}{\# Target} $$

FDR_*TDS*_ presents the FDR using an original decoy database, where #*Target* is the number of target PSMs and #*Decoy* is the number of decoy PSMs. However, target and decoy false positives are not equally likely when using small decoy database. Since the false positives from the target and decoy are not the same, we have corrected the FDR estimation by dividing it by FPRatio. The FDR using a small decoy database, FDR_*our method*_, is calculated as follows:

$$ {FDR}_{our\  method}=\frac{\# Decoy}{\# Target}\times \frac{1}{FPRatio} $$

In practice, *FPRatio* is approximated by *UPRatio* as follows.

$$ {FDR}_{our\  method}=\frac{\# Decoy}{\# Target}\times \frac{1}{UPratio} $$

## Additional file

Additional file 1: Supplementary Methods, Figures and Tables. (DOCX 1877 kb)

## Data Availability

The human data is publicly available from https://www.ebi.ac.uk/pride/archive/ using PXD002395 and *Saccharomyces cerevisiae* data is publicly available from https://chorusproject.org/anony-mous/download/experiment/-8823069691100997209.

## Abbreviations

Da: Dalton
DBRatio: Ratio of decoy to target database sizes
FDR: False discovery rate
FPRatio: Ratio of decoy to target false positive
HCD: Higher-energy collisional dissociation
MS/MS: Tandem mass spectrometry
NTT: Number of tryptic termini
PSM: Peptide spectrum match
TDS: Target-decoy search strategy
UPRatio: Ratio of decoy to target unique peptides
