# Supplementary material for: Target-small decoy search strategy for false discovery rate estimation
Source: BMC Bioinformatics. 2019 Aug 23;20:438. doi: 10.1186/s12859-019-3034-8 (PMC6708216; doi:10.1186/s12859-019-3034-8)
Supplement: Supplementary file 1 — Supplementary Methods, Figures and Tables. (DOCX 1877 kb) [file 12859_2019_3034_MOESM1_ESM.docx]

**Target-small decoy search strategy for false discovery rate estimation**

**Supporting Information**

Hyunwoo Kim^1^, Sangjeong Lee^2^, Heejin Park^2,*^

^1^ Research Data Hub Center, Korea Institute of Science and Technology Information, Daejeon 34141, Republic of Korea

^2^ Department of Computer Science, Hanyang University, Seoul 06978, Republic of Korea

Email:

Hyunwoo Kim: [pardess@kisti.re.kr](mailto:pardess@kisti.re.kr)

Sangeong Lee: [othertics@hanyang.ac.kr](mailto:othertics@hanyang.ac.kr)

Heejin Park: hjpark@hanyang.ac.kr

*Corresponding author.

Heejin Park, Email: [hjpark@hanyang.ac.kr](mailto:hjpark@hanyang.ac.kr)

**Supplementary Methods**

There are several methods to construct a decoy database.

**Reverse method:** Reverse method is to construct a decoy database by reversing of each protein in target database. This method maintains the general characteristics of target database, the reverse decoy database has same redundancy as the target database. In addition, other researchers can get same decoy database because this method create the decoy database that is fixed for the target database. EX) ABC**K**DEFG**R** 🡪 **R**GFED**K**CBA

**Pseudo reverse method:** Pseudo reverse method constructs a decoy database by reversing only the sequence between R and K in each protein in the target database. It has same characteristics as reverse method except it reverses the sequence between R and K. EX) ABC**K**DEFG**R** 🡪 CBA**K**GFED**R**

**Shuffle method:** Shuffle method is to construct a decoy database by shuffling of each protein in the target database. Unlike the reverse method, it has a stochastic characteristic. More decoy peptides are generated because target and decoy redundancy are not the same. EX) ABC**K**DEFG**R** 🡪 C**R**DGBE**K**AF

**Pseudo shuffle method:** Pseudo shuffle method constructs a decoy database by shuffling only the sequence between R and K in each protein in the target database. It has same characteristics as shuffle method except it shuffles the sequence between R and K. EX) ABC**K**DEFG**R** 🡪 BCA**K**FEGD**R**

**Assumption 1: Target and decoy databases do not overlap**

We investigated fully tryptic peptides from protein sequences in the target and decoy databases digested in silico with trypsin (the maximum number of missed cleavage sites in a peptide is 2 and the maximum peptide length is 45). Figure 2 shows the numbers of target peptides, decoy peptides, and overlapping peptides for various ratios of decoy to target database sizes. Regardless of the ratio, the portion of overlapping peptides in decoy databases whose length is 6 or more is negligible ( < 1%). Therefore, Assumption 1 is valid in our method.

**Supplementary Table 1.** The number of MS/MS spectra of the each data set

|  | Number of MS/MS spectra |
| --- | --- |
| A549 | 514,912 |
| GAMG | 625,351 |
| HEK293 | 624,108 |
| HeLa | 543,763 |
| HepG2 | 511,958 |
| Jurkat | 571,069 |
| K562 | 454,612 |
| LnCap | 639,927 |
| MCF7 | 640,530 |
| RKO | 502,079 |
| U2OS | 641,214 |
| Saccharomyces cerevisiae | 39,444 |

**Supplementary Table 2.** The number of target and decoy amino-acids in various small decoy databases for UniProt database, SwissProt database and Uniprot Saccharomyces cerevisiae database.

| UniProt | Target | Decoy | Ratio |
| --- | --- | --- | --- |
| Original | 60,206,892 | 60,206,892 | 1.00 |
| 1/2 | 60,206,892 | 30,103,861 | 0.50 |
| 1/4 | 60,206,892 | 15,051,761 | 0.25 |
| 1/6 | 60,206,892 | 10,034,810 | 0.17 |
| 1/8 | 60,206,892 | 7,525,974 | 0.13 |

| SwissProt | Target | Decoy | Ratio |
| --- | --- | --- | --- |
| Original | 24,309,466 | 24,309,466 | 1.00 |
| 1/2 | 24,309,466 | 12,154,823 | 0.50 |
| 1/4 | 24,309,466 | 6,077,395 | 0.25 |
| 1/6 | 24,309,466 | 4,052,040 | 0.17 |
| 1/8 | 24,309,466 | 3,039,302 | 0.13 |

| Saccharomyces cerevisiae | Target | Decoy | Ratio |
| --- | --- | --- | --- |
| Original | 3,089,143 | 3,089,143 | 1.00 |
| 1/2 | 3,089,143 | 1,544,695 | 0.50 |
| 1/4 | 3,089,143 | 772,379 | 0.25 |
| 1/6 | 3,089,143 | 515,356 | 0.17 |
| 1/8 | 3,089,143 | 386,241 | 0.13 |

**Supplementary Table 3.** The number of decoy protein and size of decoy database in various small decoy databases for UniProt database, SwissProt database and Uniprot Saccharomyces cerevisiae database.

| UniProt | Protein | Size(KB) |
| --- | --- | --- |
| Original | 172,300 | 76,506 |
| 1/2 | 85,892 | 38,058 |
| 1/4 | 43,715 | 19,110 |
| 1/6 | 28,620 | 12,686 |
| 1/8 | 22,151 | 9,583 |
| 1/12 | 14,357 | 6,348 |
| 1/16 | 10,844 | 4,768 |
| 1/20 | 8,533 | 3,803 |
| 1/40 | 4,391 | 1,914 |
| 1/80 | 2,098 | 947 |

| SwissProt | Protein | Size(KB) |
| --- | --- | --- |
| Original | 42,302 | 27,962 |
| 1/2 | 21,036 | 13,927 |
| 1/4 | 10,733 | 6,988 |
| 1/6 | 6,981 | 4,639 |
| 1/8 | 5,187 | 3,477 |
| 1/12 | 2,622 | 1,741 |
| 1/16 | 1,212 | 861 |

| Saccharomyces cerevisiae | Protein | Size(KB) |
| --- | --- | --- |
| Original | 6,900 | 4,034 |
| 1/2 | 3,518 | 2,019 |
| 1/4 | 1,699 | 1,001 |
| 1/6 | 1,162 | 672 |
| 1/8 | 890 | 507 |


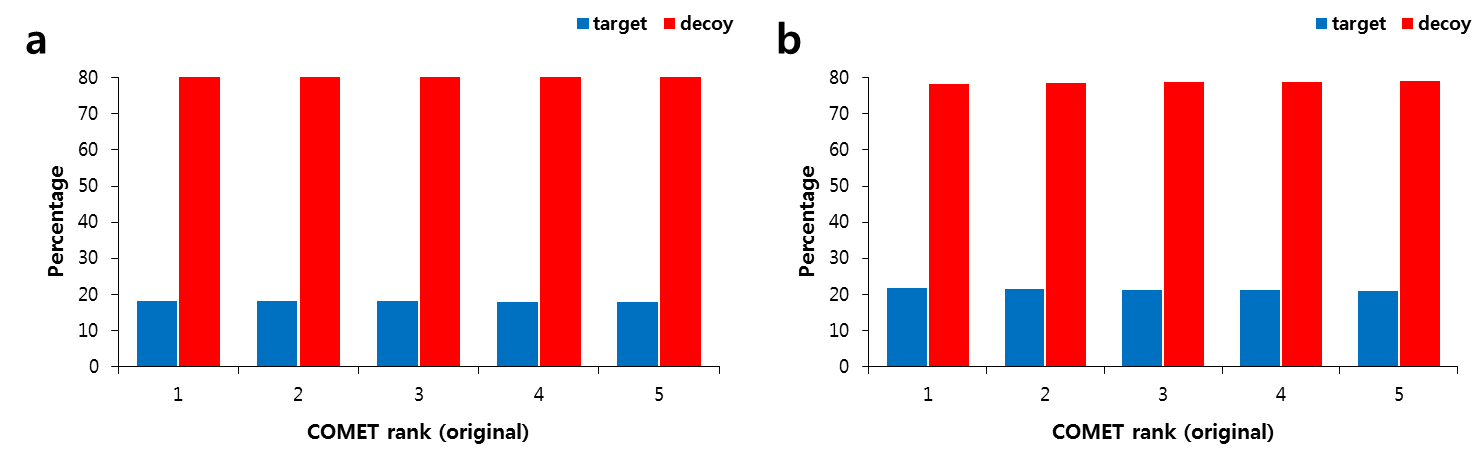


**Supplementary Figure 1. The percentages of target/decoy PSMs among the ranks 1-5 PSMs in the Comet results using the shifted HEK293 data and the UniProt database. Blue bars represent the percentage of target PSMs and red bars represent that of decoy PSMs. (a) Shuffle decoy database; (b) Pseudo shuffle decoy database.**


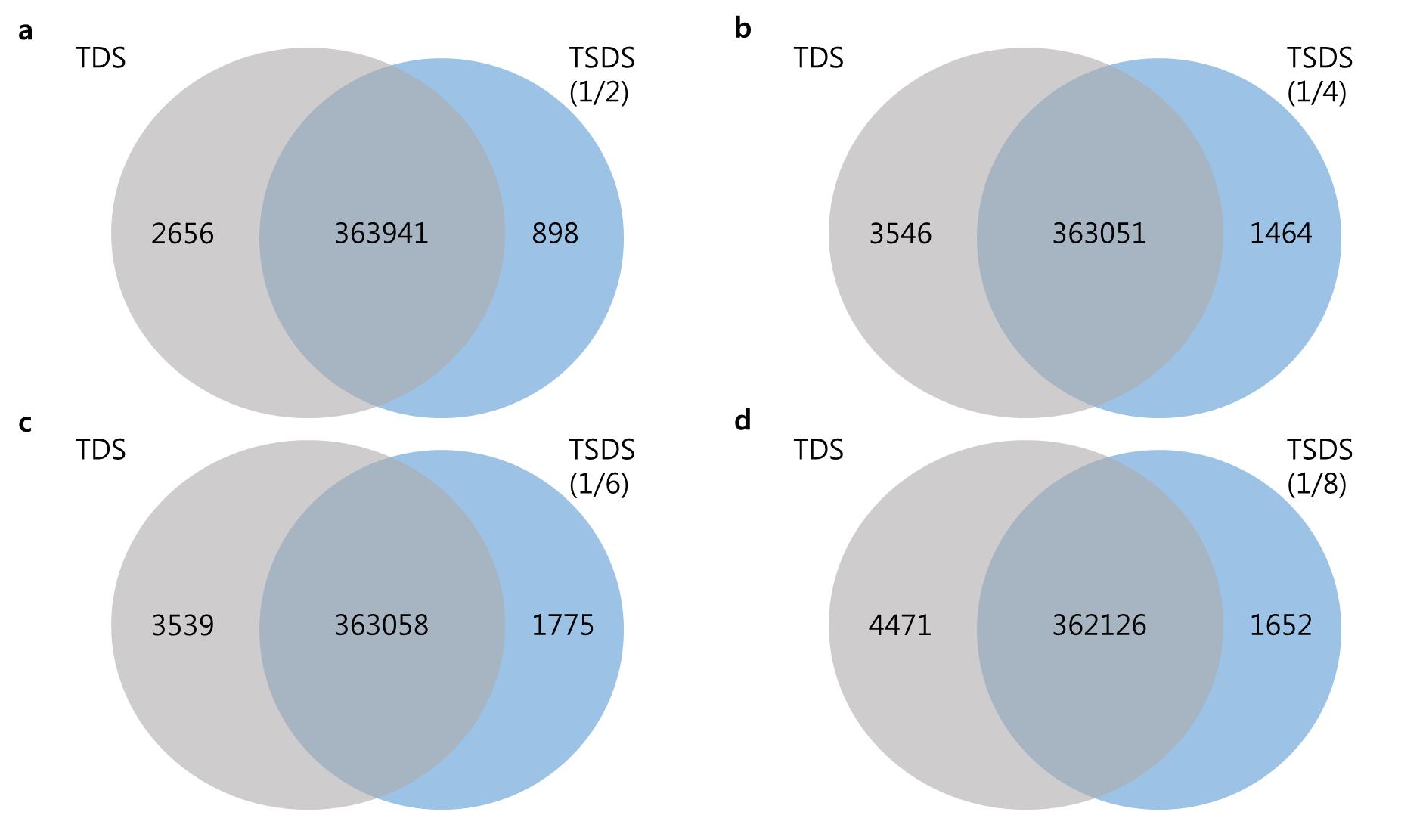


**Supplementary Figure 2. Comparison of PSMs between TDS and our method using Comet and SwissProt database. (a) 1/2 decoy database; (b) 1/4 decoy database; (c) 1/6 decoy database; (d) 1/8 decoy database.**


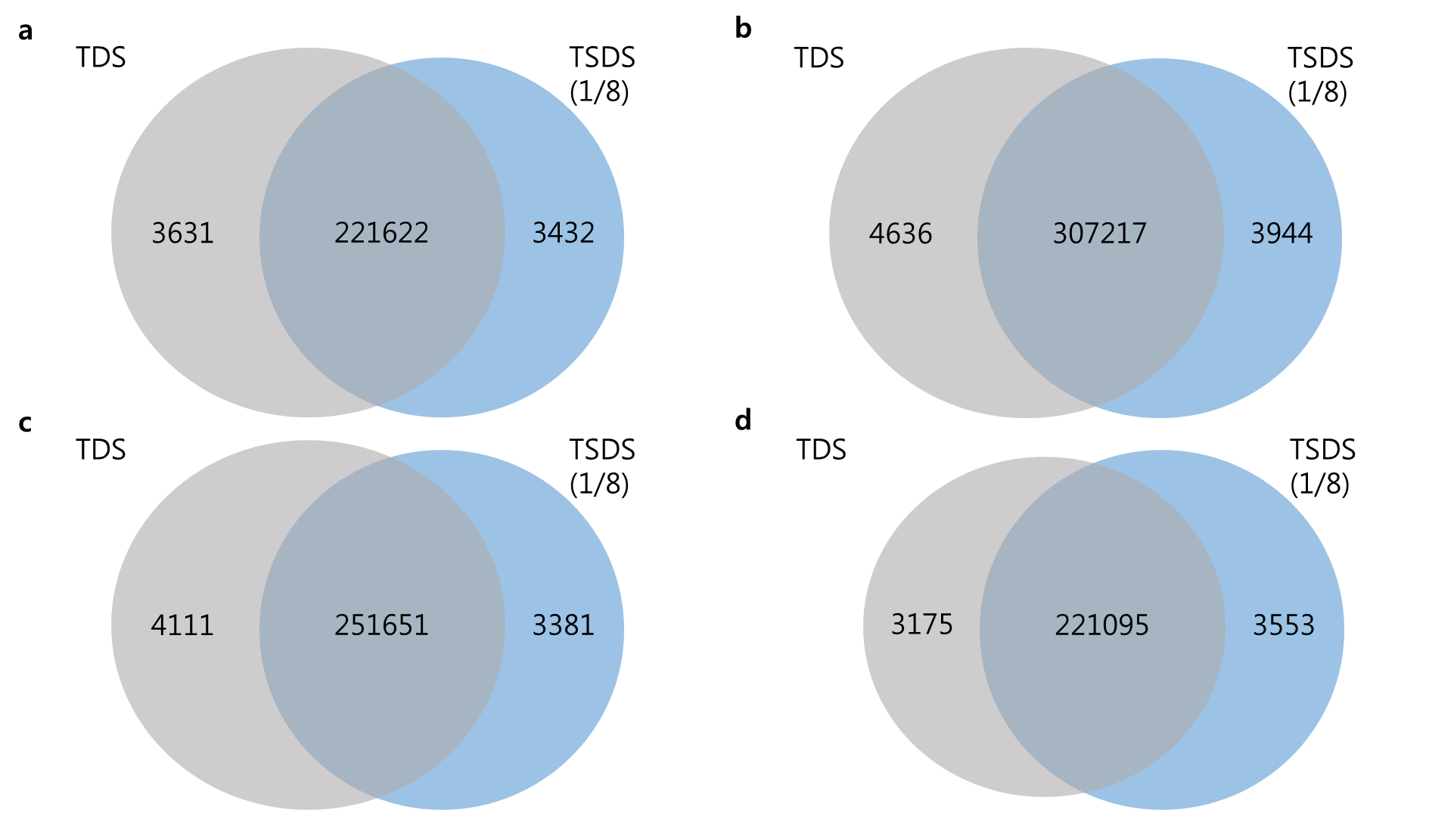

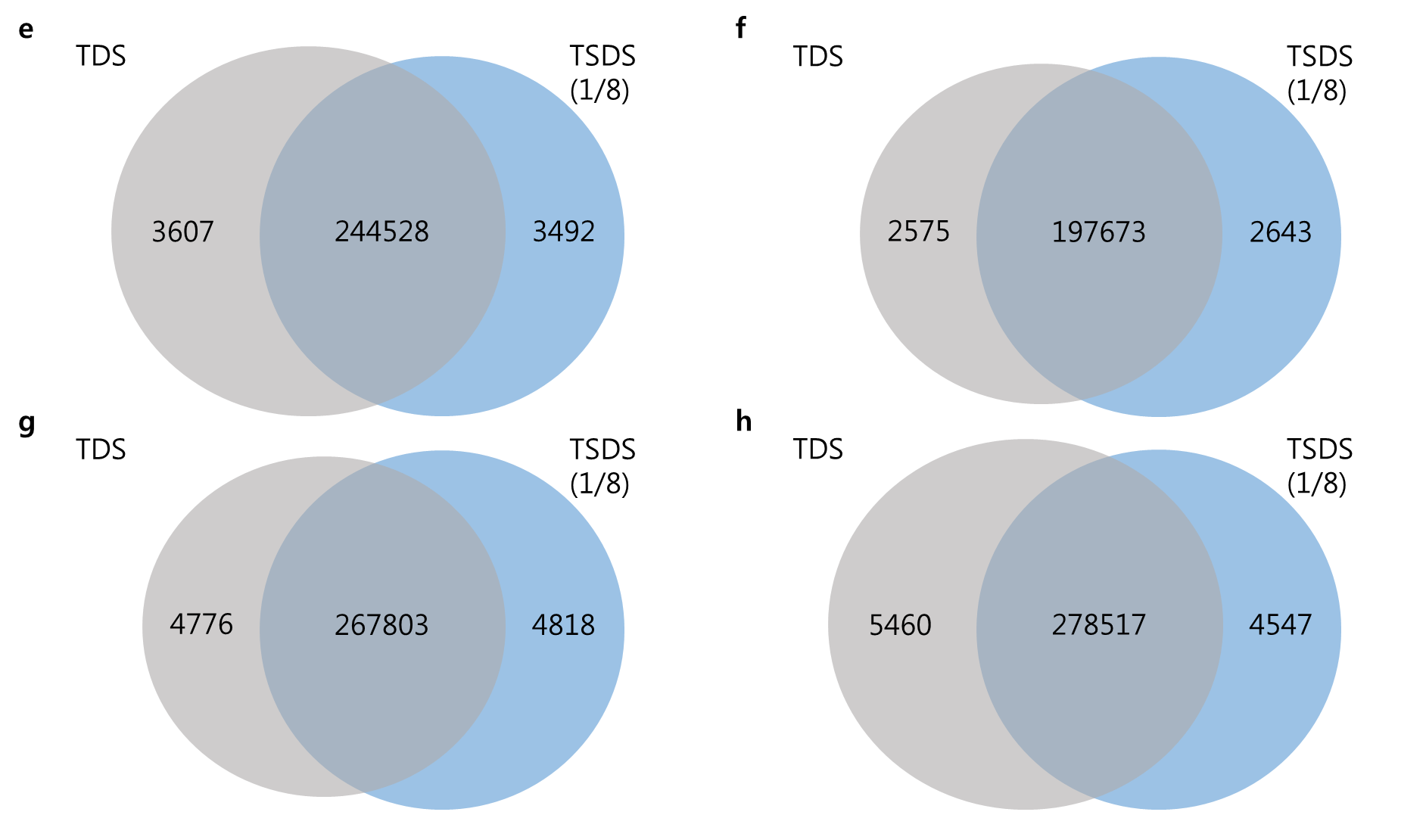

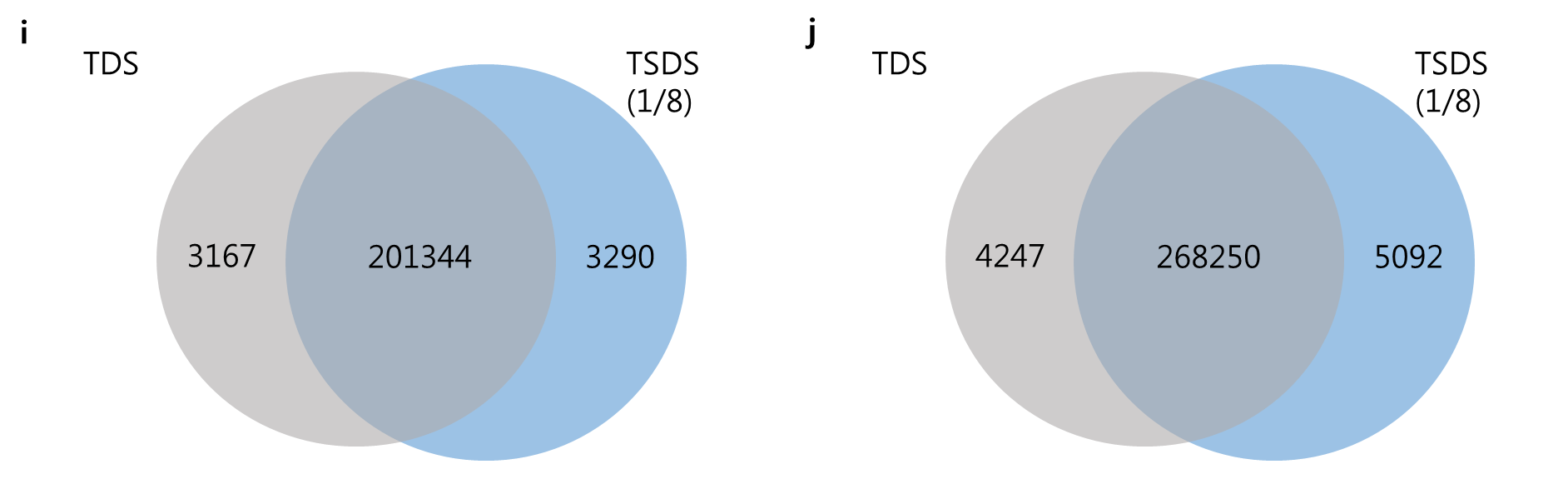


**Supplementary Figure 3. Comparison of PSMs between TDS and our method using Comet when using the 1/8 UniProt decoy database. (a) A549; (b) GAMG; (c) HeLa; (d) HepG2; (e) Jurkat; (f) K562; (g) LnCap; (h) MCF7; (i) RKO; (j)U2OS.**


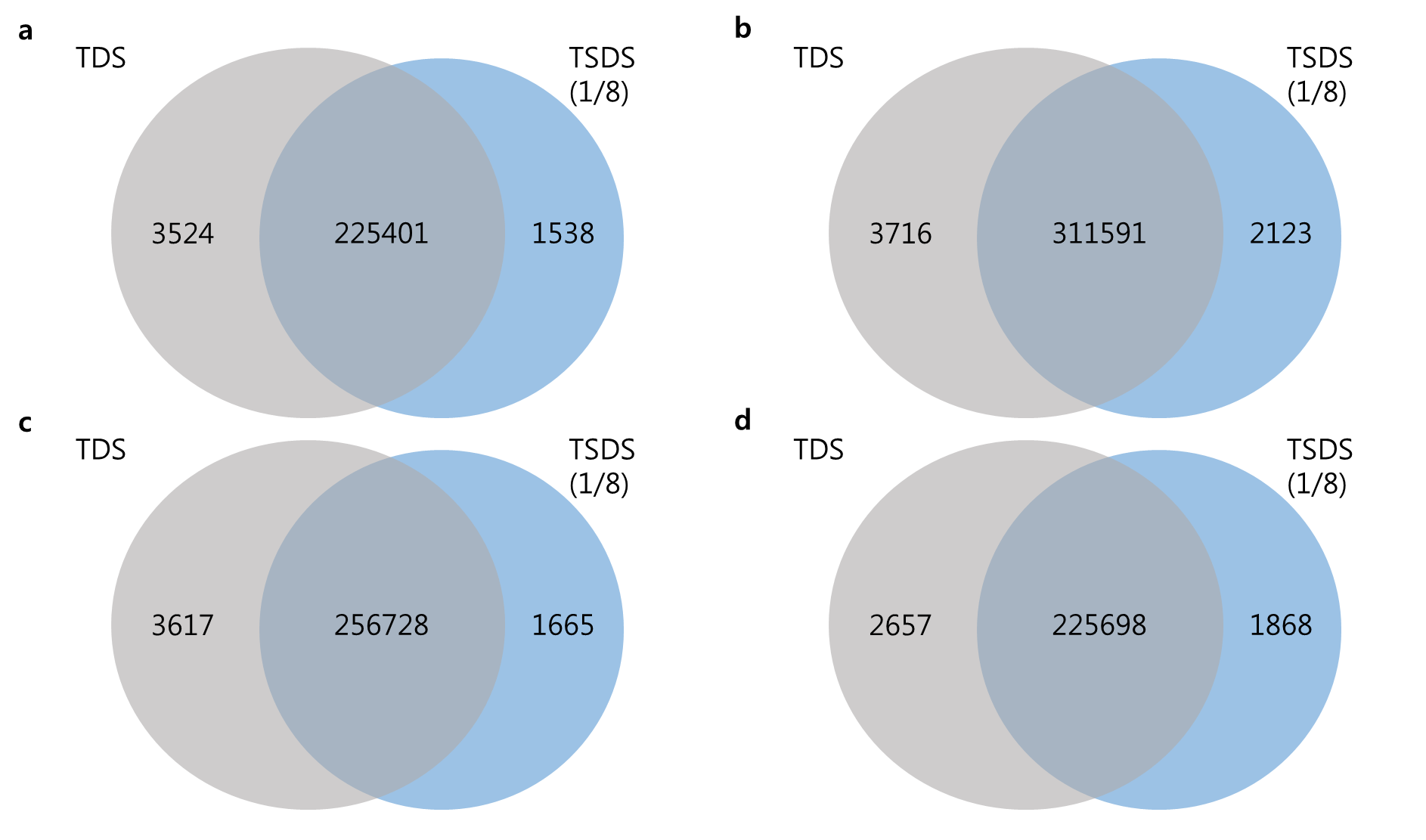

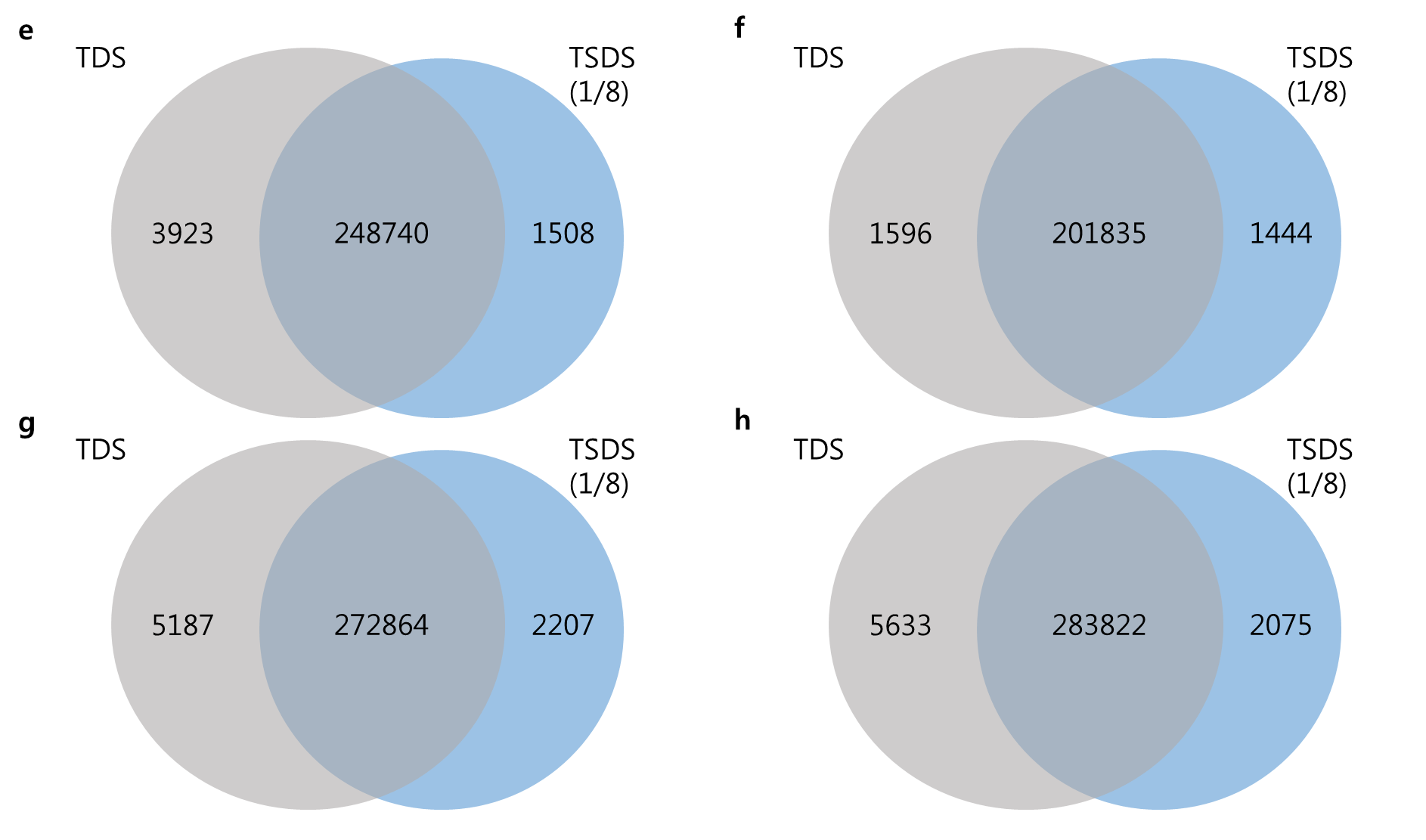

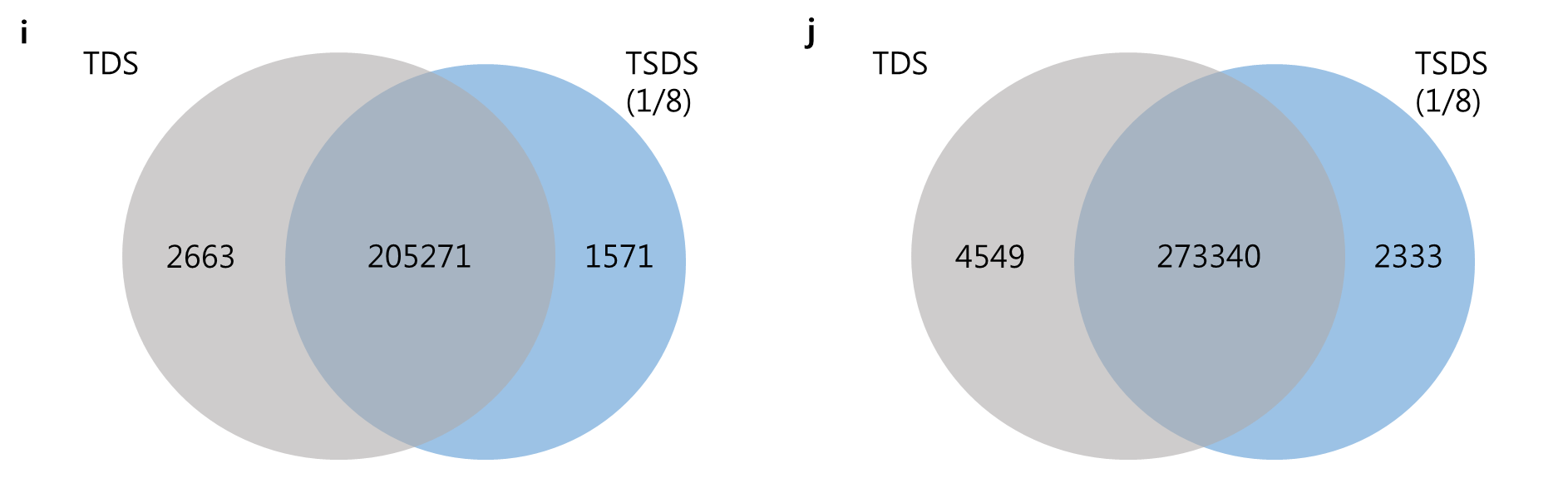


**Supplementary Figure 4. Comparison of PSMs between TDS and our method using Comet when using the 1/8 SwissProt decoy database. (a) A549; (b) GAMG; (c) HeLa; (d) HepG2; (e) Jurkat; (f) K562; (g) LnCap; (h) MCF7; (i) RKO; (j)U2OS.**

**
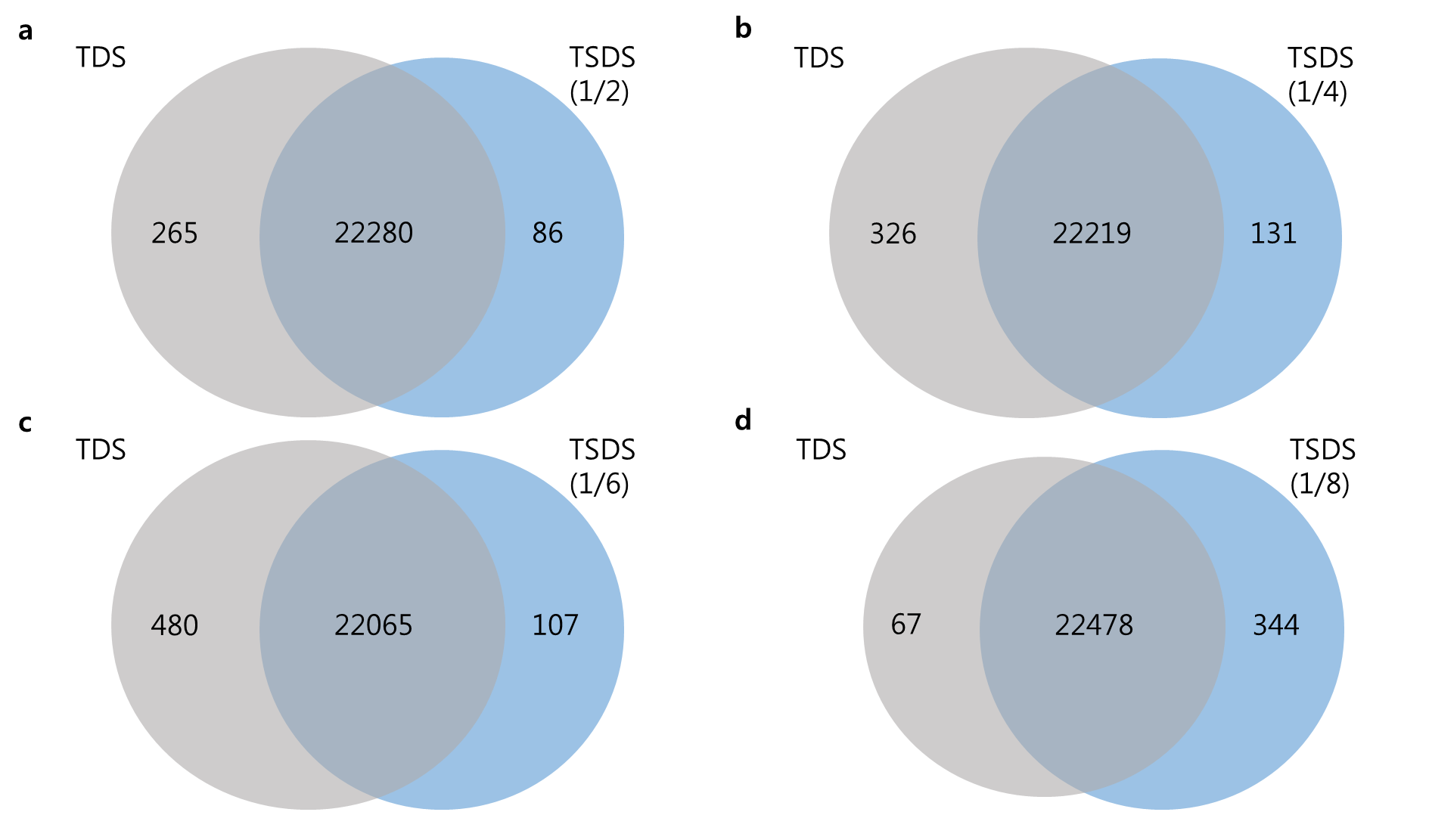
**

**Supplementary Figure 5. Comparison of PSMs between TDS and our method using Comet. (a) 1/2 decoy database; (b) 1/4 decoy database; (c) 1/6 decoy database; (d) 1/8 decoy database. Using the UniProt Saccharomyces cerevisiae databases.**

**
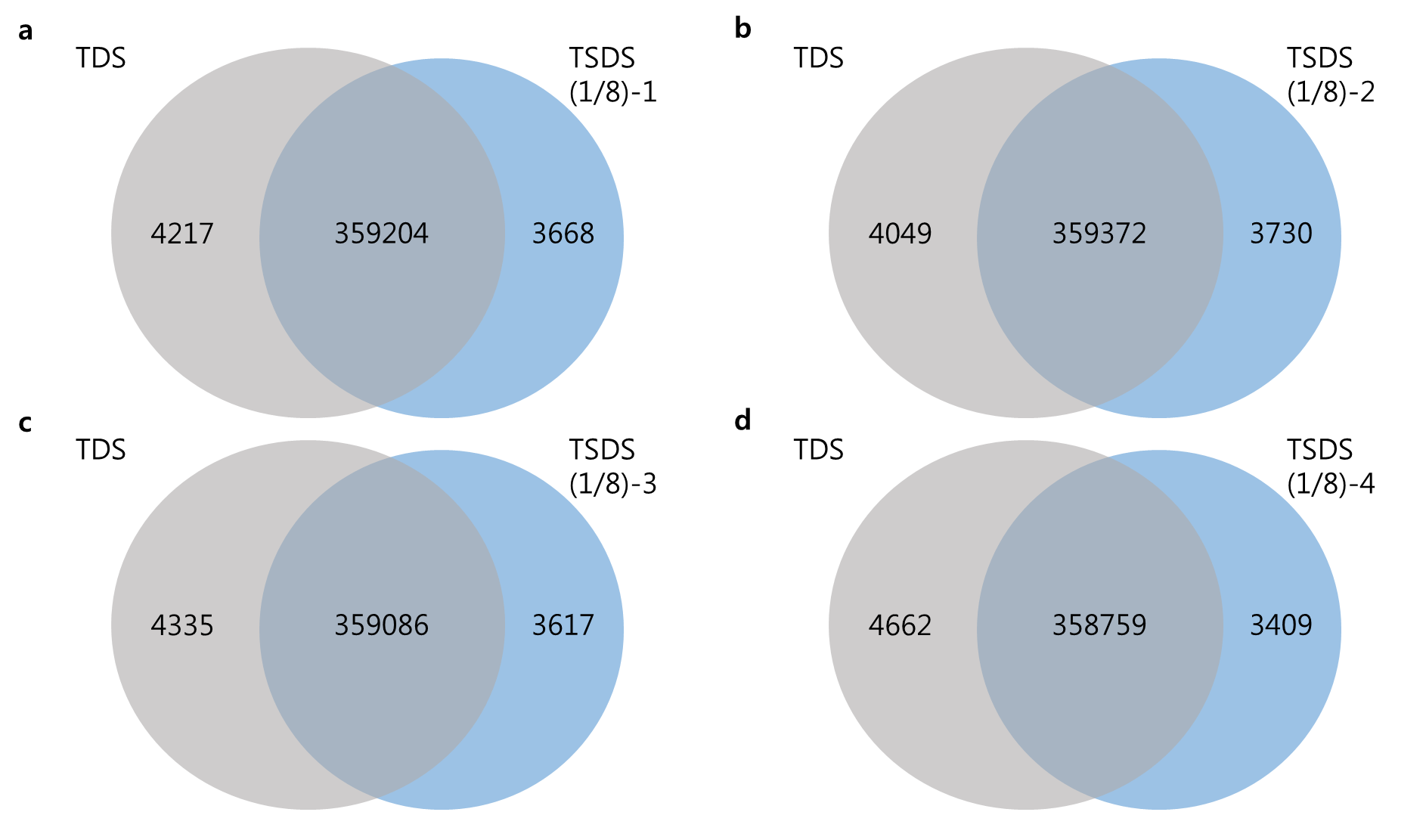
**

**Supplementary Figure 6. Comparison of PSMs between TDS and our method (random decoy database) using Comet. (a) 1/8 decoy database; (b) 1/8 decoy database; (c) 1/8 decoy database; (d) 1/8 decoy database. Using the UniProt databases.**


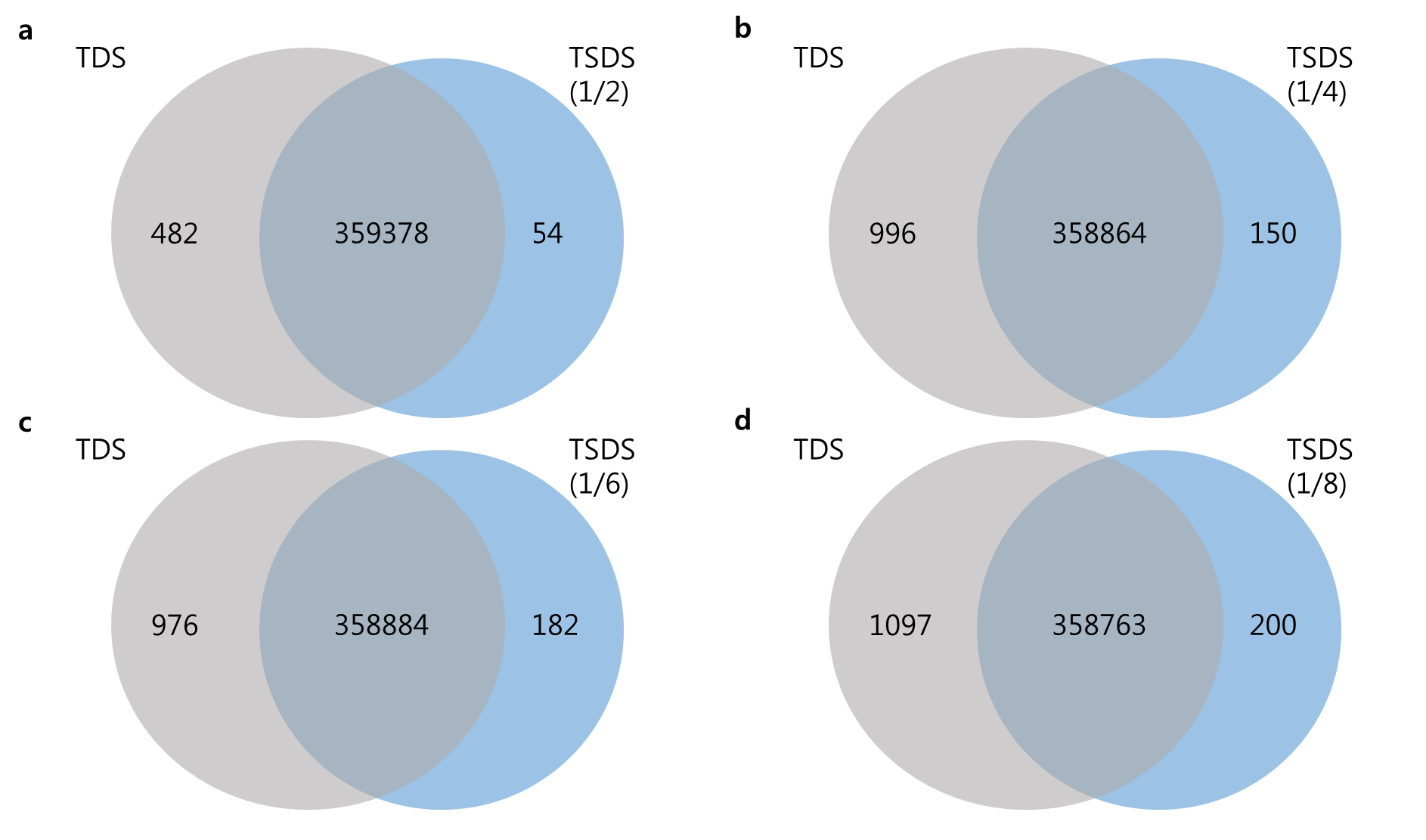


**Supplementary Figure 7. Comparison of PSMs between TDS and our method using MS-GF+ and UniProt database. (a) 1/2 decoy database; (b) 1/4 decoy database; (c) 1/6 decoy database; (d) 1/8 decoy database.**


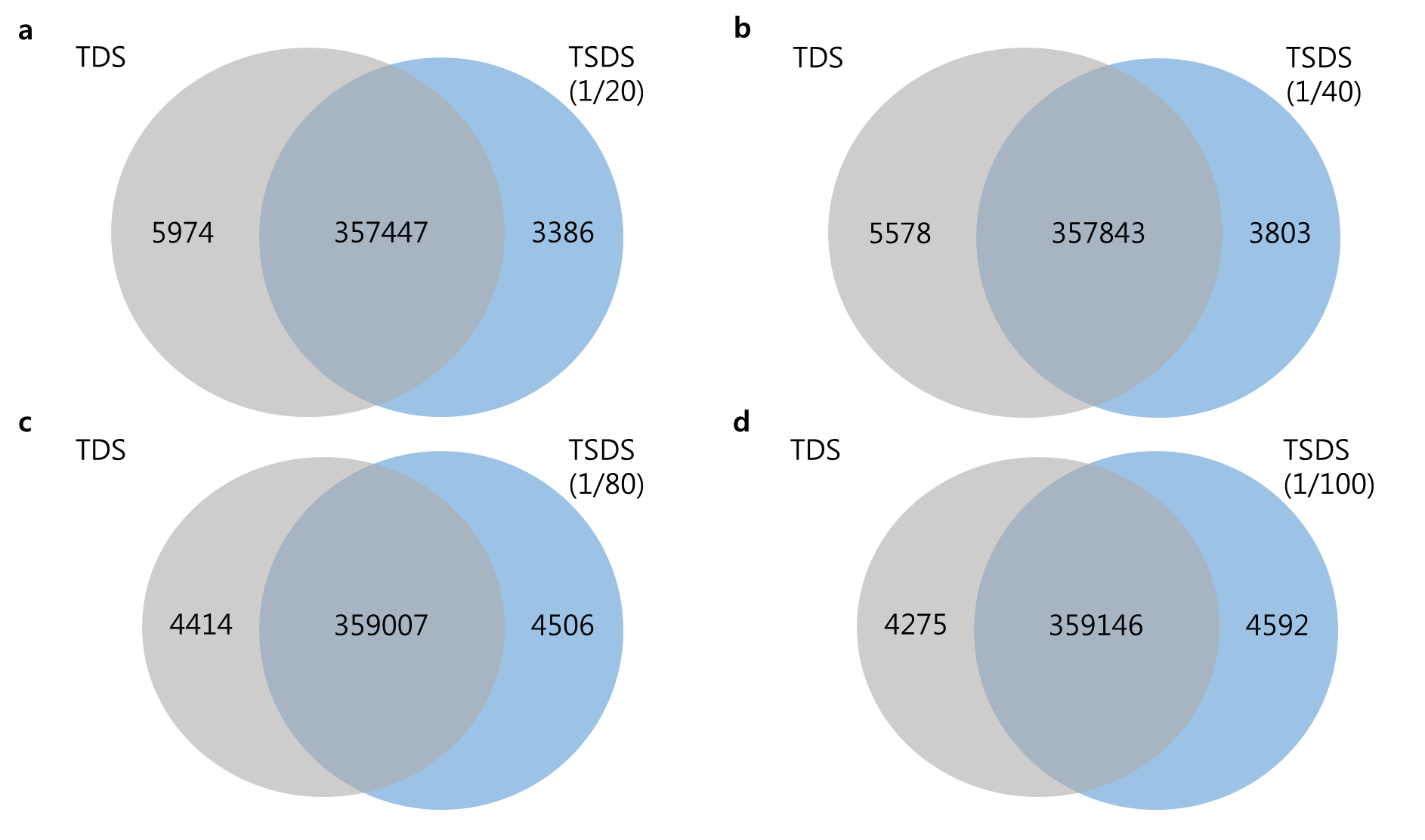


**Supplementary Figure 8. Comparison of PSMs between TDS and our method using Comet and UniProt database. (a) 1/20 decoy database; (b) 1/40 decoy database; (c) 1/80 decoy database; (d) 1/100 decoy database.**


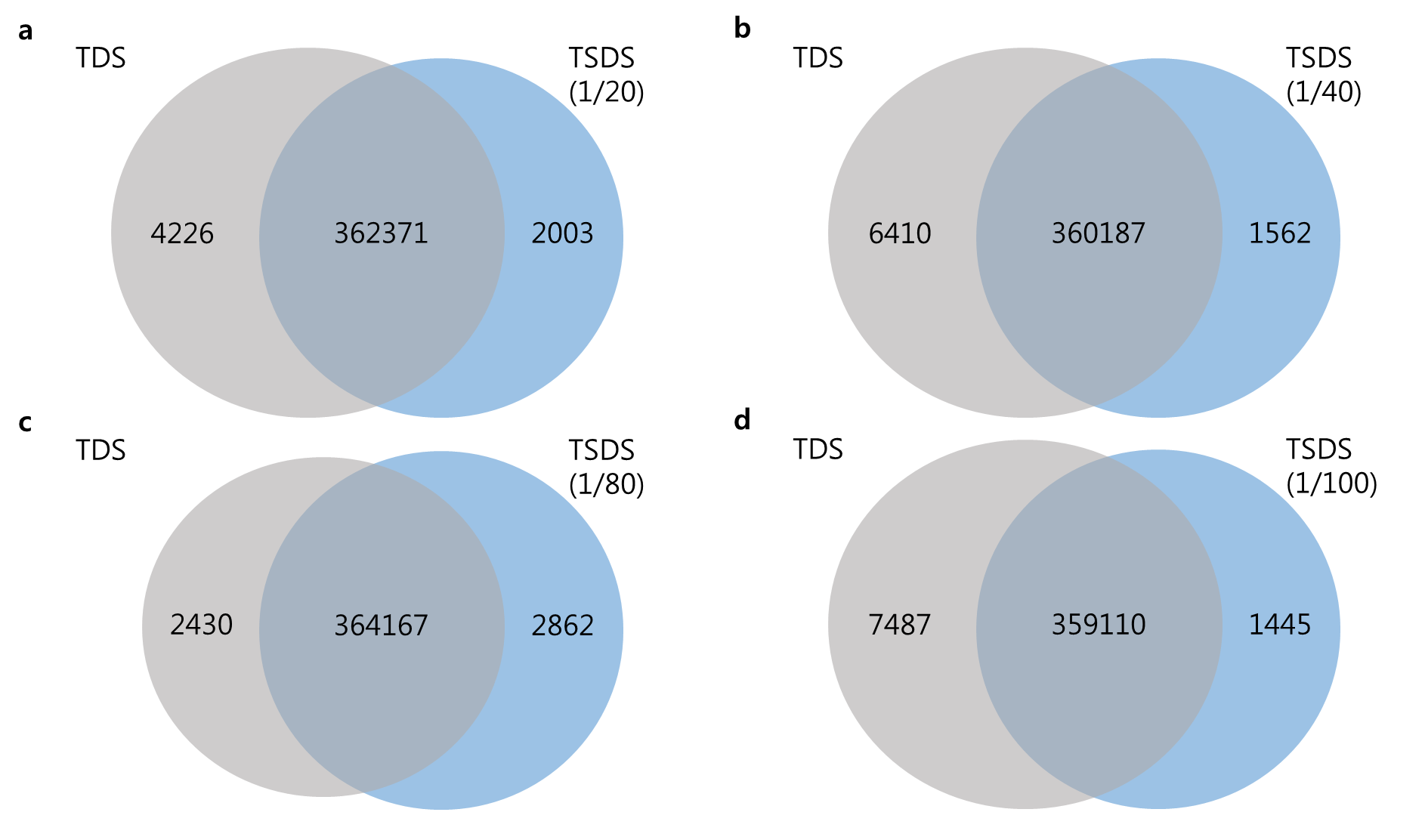


**Supplementary Figure 9. Comparison of PSMs between TDS and our method using Comet and SwissProt database. (a) 1/20 decoy database; (b) 1/40 decoy database; (c) 1/80 decoy database; (d) 1/100 decoy database.**

**
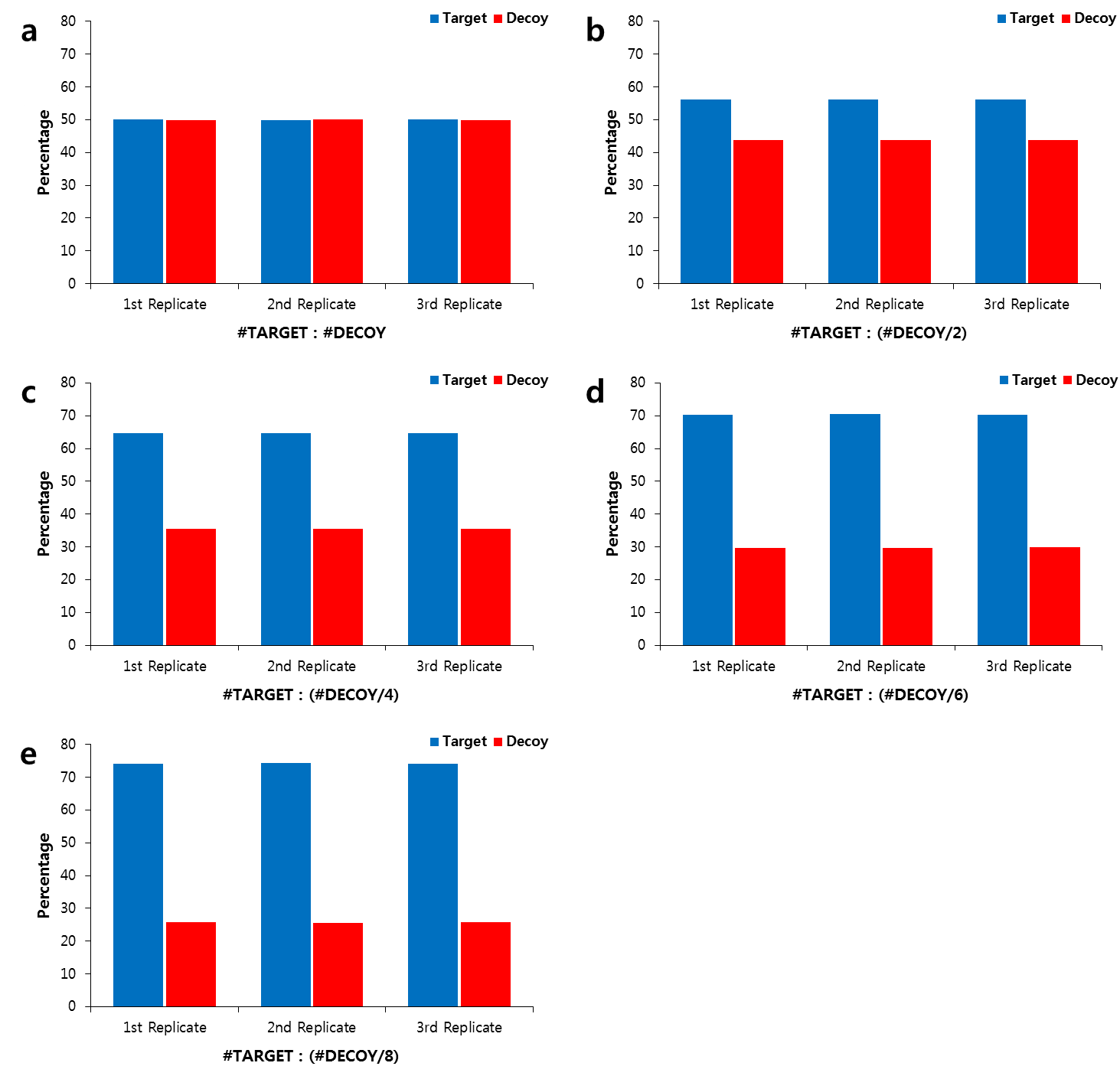
Supplementary Figure 10. The percentage of rank 1 PSMs on each replicate in the Comet results using the UniProt database. Blue bars represent the percentage of target PSMs and red bars represent the percentage of decoy PSMs. (a) Original decoy database; (b) 1/2 decoy database; (c) 1/4 decoy database; (d) 1/6 decoy database; (e) 1/8 decoy database.**


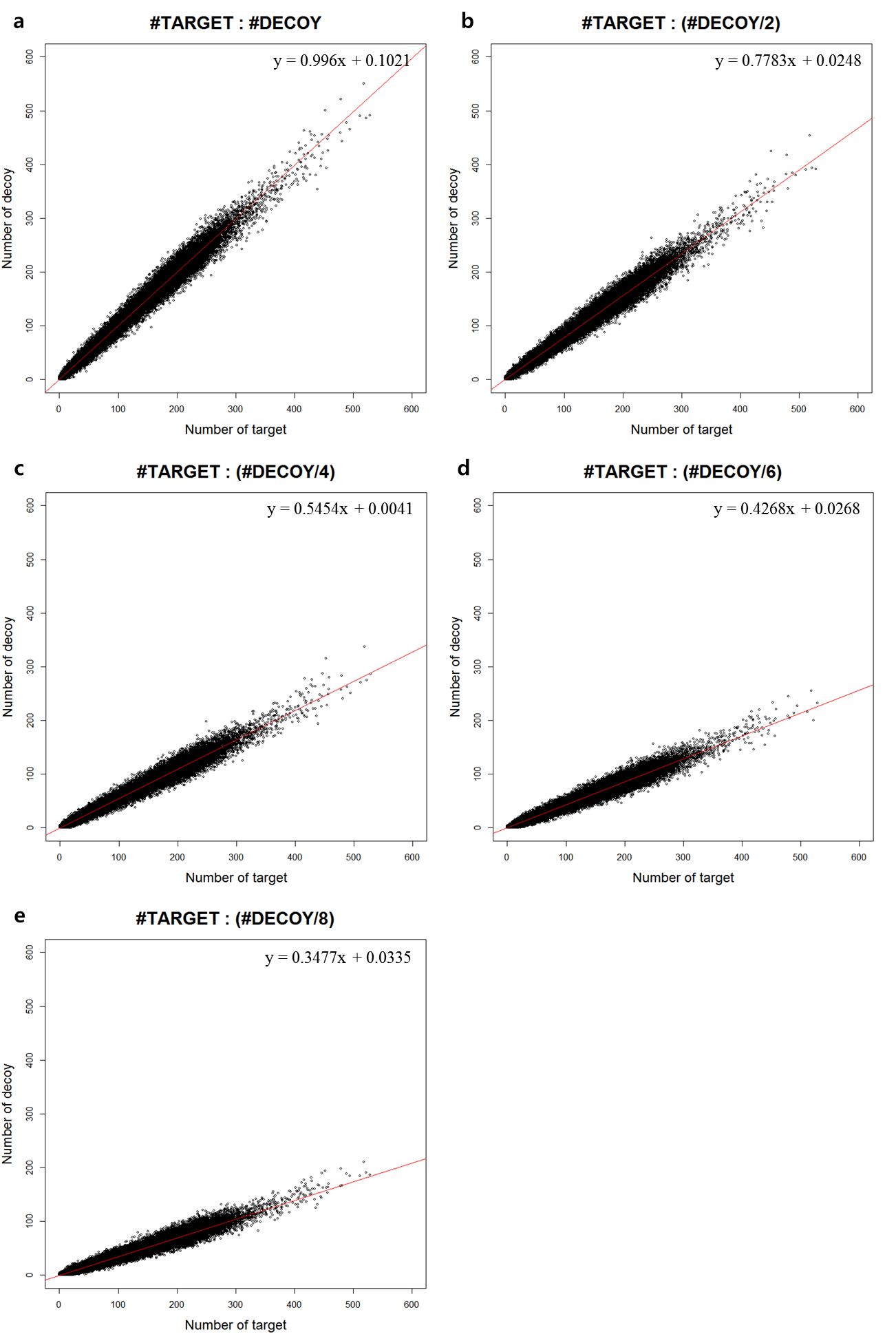


**Supplementary Figure 11. The number between target and decoy peptides were compared for every mass using the UniProt database. As the decoy database decreases, the slope decreases at a constant rate. (a) Original decoy database; (b) 1/2 decoy database; (c) 1/4 decoy database; (d) 1/6 decoy database; (e) 1/8 decoy database.**


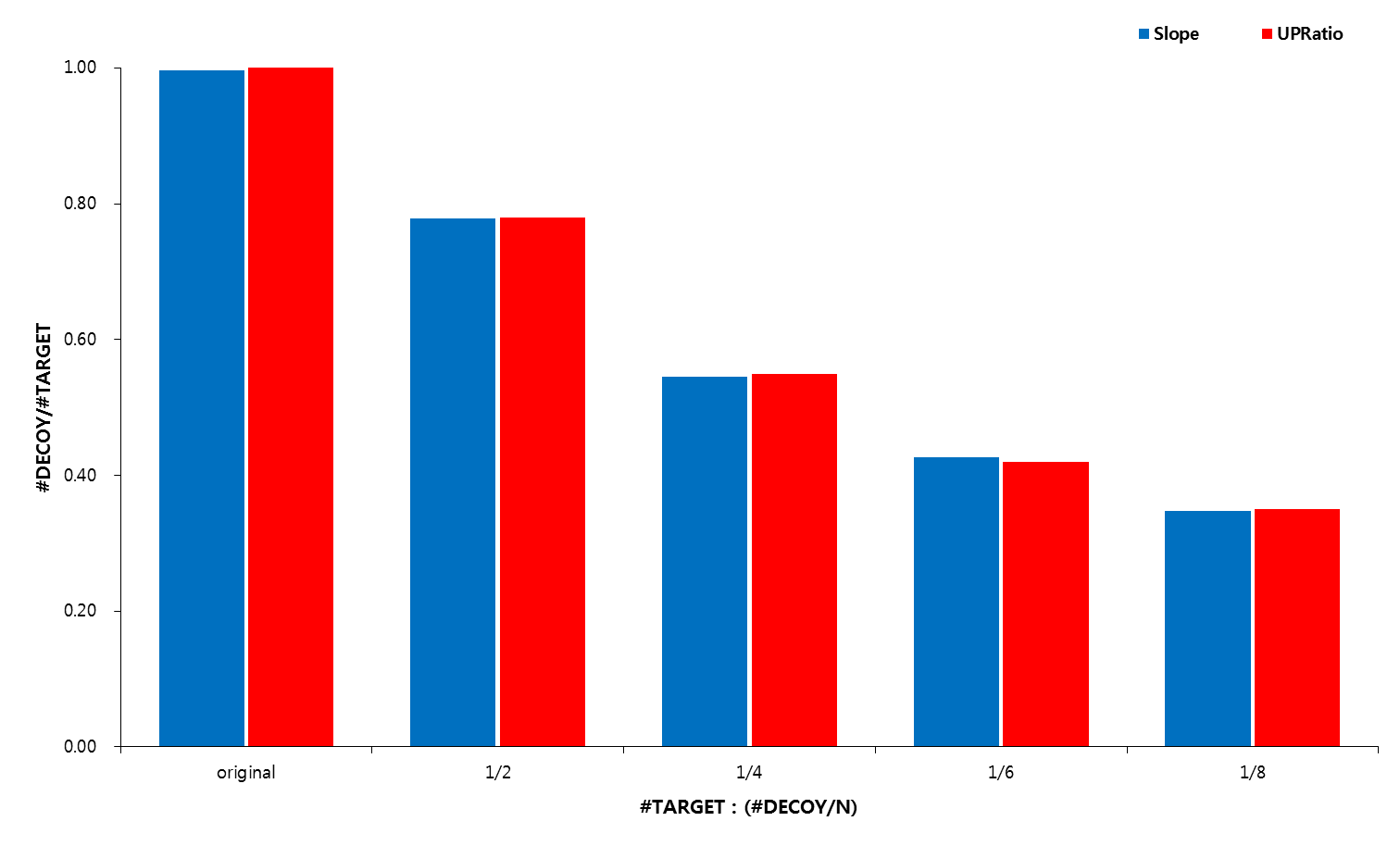


**Supplementary Figure 12. Comparison of ratios between slope of Supplementary Figure 3 (blue bars) and UPRatio (red bars).**
